# No association of childhood maltreatment experiences and attention to facial emotions in men

**DOI:** 10.1038/s41598-026-65088-1

**Published:** 2026-08-05

**Authors:** Dennis Hoepfel, Jakob Jantz, Vivien Günther, Katja Koelkebeck, Anette Kersting, Thomas Suslow

**Affiliations:** 1 Department of Psychosomatic Medicine and Psychotherapy, University of Leipzig Medical Center, Leipzig, Germany; 2 Department of Psychiatry and Psychotherapy, Medical School and University Medical Center OWL, Protestant Hospital of the Bethel Foundation, Bielefeld University, Bielefeld, Germany

**Keywords:** Childhood maltreatment, Maltreatment subtypes, Facial emotions, Attention, Childhood abuse, Childhood neglect, Free-viewing task, Diseases, Psychology, Psychology

## Abstract

A recent study investigated the relationship between childhood trauma and attention to emotions as a function of type and severity of maltreatment in a sample of women. It was found that high levels of physical and emotional abuse severity were associated with a diminished attentional preference for happy faces. Given that men exhibit distinct patterns of childhood maltreatment and often show lower accuracy and attention toward facial emotions compared to women, investigating these relationships specifically in male populations is warranted. In the present study, we examined the relationship between childhood trauma and attention to emotions in a sample of men. Gaze behavior of 98 men with a history of childhood trauma was analyzed in a free-viewing task. Pairs of faces with an emotional (happy, surprised, angry, disgusted, fearful, and sad) and a neutral face were shown for 5 s. An attentional bias score was calculated by subtracting the total fixation duration on the neutral face from that on the emotional face. To assess childhood maltreatment experiences, the Childhood Trauma Questionnaire was employed. Linear mixed models were used to investigate research questions. The attentional bias scores exhibited high reliability. For all emotion categories, participants looked longer at the emotional compared to the neutral face. The highest attentional preference was found for happy faces. Results indicated neither a significant main effect of childhood maltreatment type, nor a significant interaction effect between childhood maltreatment type and emotion category. Similarly, no main effect or interaction involving overall childhood maltreatment severity was found. The present findings reveal no significant relationship between childhood experiences of maltreatment (including emotional, sexual, and physical abuse, as well as emotional neglect) and visual attention to facial emotions in men. Our data do not support the hypothesis that early life trauma may lead to alterations in attentional processing of emotional facial expressions in men.

## Introduction

Research has shown that experiences of childhood abuse and neglect have persistent negative effects on physical and mental health^1,2^. Different forms of childhood abuse (e.g., physical, emotional, and sexual) and neglect (physical, and emotional) may have different effects on the type and severity of psychopathological symptoms in adulthood^3,4^. Repeated or prolonged experiences of violence and maltreatment by caregivers could lead to an increased sensitivity to threat-related stimuli^5,6^.

The dot-probe task^7^ has been frequently administered in research on childhood adversity to assess attention biases. Studies on types of childhood maltreatment and negative attentional biases based on the dot-probe procedure have produced inconsistent results. In a sample of maltreated children, physical maltreatment but not neglect was related to an attentional bias away from angry faces^8^. Kelly et al.^9^ observed also an attentional bias away from angry faces in maltreated children, but these children were primarily characterized by neglect and emotional abuse. Günther et al.^10^ found a positive association of physical neglect and emotional abuse with an attentional bias towards sad faces in depressed patients. Blauth and Iffland^11^ reported associations of emotional abuse with increased attention to angry faces and associations of emotional neglect with attentional avoidance of angry faces in a community sample of adults. Hori et al.^12^ did not reveal relations between subtypes of childhood maltreatment and attentional biases either toward or away from negative words in healthy women. These mixed results could be due to differences in experimental stimuli and procedures and study participants’ biological sex, age, and psychopathological status. Another factor that could explain the inconsistent findings is the low reliability of the dot-probe task. Poor reliability of attention bias indices (reaction time- and accuracy-based scores) was observed for different versions of the dot-probe task^13,14^. Reliable measures of attention are necessary to advance our understanding of the attention processes that might be involved in childhood trauma and psychopathology^15^.

Compared to reaction-time tasks such as the dot probe task, eye-tracking systems allow a direct and continuous measurement of visual attention^16^. For free viewing tasks, indices of attentional bias based on fixation duration show adequate to good reliability^17,18^. Only a few eye-tracking studies have investigated the relationship between childhood maltreatment experiences and attention to emotions in adulthood. Bodenschatz et al.^19^ observed that overall severity of childhood maltreatment exposure was linked to an avoidance of angry and sad facial expressions during free viewing in a mixed-sex sample of depressed patients. Similarly, in a sample of healthy women, Hoepfel et al.^20^ found evidence of an association between severity of adverse childhood experiences and attentional avoidance of disgusted facial expressions at a late stage of perception. In a recent investigation focusing on the subtypes of childhood maltreatment^21^, high levels of physical and emotional abuse went along with decreased attention to positive facial expressions, and, in addition, severe levels of emotional abuse were related to less attention to disgusted faces. The latter findings suggest that attention to social emotional information in adulthood might depend on the type and severity of childhood trauma in women.

In the present eye-tracking study, the association between subtypes of childhood maltreatment and allocation of attention to six facial emotions (happiness, surprise, anger, disgust, fear, and sadness) was investigated in a sample of men using a free-viewing task. We recruited men with experiences of childhood abuse (physical, emotional, and/or sexual) and/or childhood neglect (physical and/or emotional). Our study could help to specify which type and at what level adverse childhood experiences are linked to attentional biases toward or away from emotional facial expressions in men. None of the previous studies on the topic has examined exclusively men (or boys). The majority of previous investigations included exclusively^12,20,21^ or predominantly women (or girls) as study participants^8,10,11^. Against the background that men show distinct patterns of childhood maltreatment^22,23^ and manifest lower recognition accuracy of and attention toward facial emotions compared to women^24,25^, examining these relationships specifically in male populations is warranted. In our investigation, we also examined the relation of overall childhood maltreatment severity with attention allocation to facial emotions. The analysis of eye-movements was based on the time participants’ gaze was fixated on the emotional compared to the neutral face as an index of attentional preference. Due to a scarcity of research involving male participants, our hypotheses were derived from a prior eye-tracking study on women using the same experiment^21^, despite the aforementioned sex-specific patterns in emotion perception. Based on the findings of our previous eye-tracking study with female individuals^21^ we expected that at high levels of physical and emotional abuse, attentional preference for positive compared to other emotional faces would be diminished. In addition, it was hypothesized that at a severe level of emotional abuse a bias away from disgusted faces should occur.

## Methods

### Participants

The final sample consisted of ninety-eight men with a mean age of 25.17 years (SD: 4.29; range: 18 to 35). Study participants with maltreatment experiences during childhood were recruited through traditional methods such as posting recruitment ads on noticeboards of canteens and libraries and social media. Study participants were native German speakers or spoke German since the age of six; only a few participants had a slightly lower language level. In the present study, the maximum age of participants was 35 years, while in our previous study^21^ it was 30 years. Participants were screened for multiple exclusion criteria: self-reported current or previous psychiatric or neurological illnesses, and compromised vision. To exclude the presence of a major depressive episode we asked whether participants lost interest or enjoyment in activities over a two-week period in the last month or whether they felt sad, hopeless, or empty most of the day for a two-week period in the past month. All participants reported during the screening interview to have experienced at least one childhood maltreatment form. This was confirmed by the CTQ data for all participants but one: according to the severity classification of Bernstein and Fink^26^ ninety-seven study participants had at least one type of childhood maltreatment experience at level 2 “low to moderate”. Most study participants (*n* = 58) were university students. The other participants were working (*n* = 22), in vocational training (*n* = 4), or unemployed (*n* = 13) (one participant did not provide information). Before partaking in our study, subjects gave written informed consent. The study was conducted in accordance with the principles of the Declaration of Helsinki^27^. Ethical approval for this project was obtained from the ethics committee of the Medical School at the University of Leipzig (DE/EKSN40). Clinical trial number: not applicable.

### Self-report questionnaires and tests

The Childhood Trauma Questionnaire (CTQ^26^; German version^28^) was used to assess experiences of childhood maltreatment. The CTQ consists of the subscales emotional neglect, physical neglect, emotional abuse, physical abuse, and sexual abuse (with 5 items respectively). Items are rated on a 5-point scale. In the present study, the subscale physical neglect was not included in our analysis as it had poor internal consistency in our sample (Cronbach’s alpha: 0.52). Cronbach’s alpha coefficients for all CTQ scales are shown in Table 1. Klinitzke et al.^29^ reported also low internal consistency for physical neglect and cautioned against the use of the scale.

**Table 1:** Descriptive statistics of and correlations between self-report scales and tests (*N* = 98) with Cronbach’s alpha for self-report questionnaires.

Variable	1	2	3	4	5	6	7	8	9	10	11	α
1. CTQ												0.85
2. CTQ-PA	0.73***											0.78
3. CTQ-EA	0.74***	0.36***										0.75
4. CTQ-SA	0.49***	0.24*	0.28**									0.90
5. CTQ-EN	0.67***	0.31**	0.40***	0.04								0.75
6. ERQ-Re	− 0.17	− 0.10	− 0.04	− 0.02	− 0.26**							0.82
7. ERQ-Su	0.21*	0.06	0.18	0.05	0.23*	− 0.02						0.75
8. STAI T	− 0.01	− 0.09	0.13	0.05	− 0.07	− 0.12	0.11					0.90
9. BDI-II	0.26*	0.04	0.29**	0.06	0.26*	− 0.29**	0.37***	0.37***				0.89
10. PSS-10	0.17	0.02	0.14	0.04	0.24*	− 0.42***	0.19	0.46***	0.64***			0.87
11. TAS-20	0.06	0.10	0.02	0.00	0.05	− 0.16	0.35***	0.08	0.35***	0.38***		0.85
12. MWT-B	0.06	0.02	0.03	− 0.11	0.11	− 0.10	− 0.06	− 0.09	− 0.07	− 0.05	− 0.11	-
	**1**	**2**	**3**	**4**	**5**	**6**	**7**	**8**	**9**	**10**	**11**	**12**
Mean	53.95	8.68	12.58	6.34	16.83	26.86	16.04	46.95	14.86	30.17	47.00	106.57
SD	13.06	4.58	4.76	3.48	3.75	7.44	4.92	5.23	9.49	7.03	11.81	10.07

* *p* < .05 (two-tailed), ** *p* < .01 (two-tailed), *** *p* < .001 (two-tailed).

CTQ: Childhood Trauma Questionnaire; CTQ-PA: scale physical abuse; CTQ-EA: scale emotional abuse; CTQ-SA: scale sexual abuse; CTQ-EN: scale emotional neglect; ERQ-Re: scale cognitive reappraisal of the Emotion Regulation Questionnaire; ERQ-Su: scale expressive suppression of the Emotion Regulation Questionnaire; STAI T: State Trait Anxiety Inventory, trait version; BDI-II: Beck Depression Inventory; PSS-10: Perceived Stress Scale; TAS-20: 20-Item Toronto Alexithymia Scale; MWT-B: Mehrfachwahl-Wortschatz-Intelligenztest version B, intelligence quotient.

We used the Beck Depression Inventory (BDI-II^30^, German version^31^), which comprises 21 items, to assess the presence and severity of depressive symptoms during the preceding two weeks.

The trait version of the State-Trait Anxiety Inventory (STAI^32^, German version^33^) was administered to measure dispositional anxiety. The STAI consists of 20 items.

We administered the Perceived Stress Scale (PSS-10^34^, German version^35^) to assess the extent to which participants experience their lives as stressful during the last month. The PSS-10 consists of 10 items.

The personality trait alexithymia was measured using the 20-Item Toronto Alexithymia Scale (TAS-20^36^, German version^37^).

Habitual engagement of expressive suppression and cognitive reappraisal were measured with the Emotion Regulation Questionnaire (ERQ^38^, German version^39^). The questionnaire contains 10 self-referential statements that relate to dealing with positive and negative feelings.

The Mehrfachwahl-Wortschatz-Intelligenztest version B (MWT-B^40^) was used to measure participants’ intelligence. The MWT-B is a performance test with no time restrictions that consists of 37 items. MWT-B sum scores show high correlations with global IQ in healthy adults^41^.

### Free-viewing task: stimuli and procedure

We selected 140 faces of twenty models (10 female and 10 male) from the Karolinska Directed Emotional Faces (KDEF^42^). A neutral expression and six emotional expressions (anger, fear, disgust, sadness, surprise, and happiness) were chosen for each model. Pairs of faces were presented; an emotional face combined with a neutral face of the same model. Combining the six emotional expression images with the neutral expression image for each of the 20 models yielded 120 trials. Positions of faces were counterbalanced. All stimuli were shown in their original color on a grey background.

Each trial of the free-viewing task began with a fixation cross at the center of the screen. When participants fixated on it for 1000ms, the fixation cross was replaced with a pair of faces, which was displayed for 5000ms. Immediately after that the next trial was initiated. Trials were presented in a fixed random order. Participants were told to freely view the pictures on the computer screen. They were seated approximately 40 cm from the monitor. Ceiling lighting produced a stable illuminance condition. The laboratory was protected from sunlight by closed roller blinds.

### Eye-tracking apparatus

The PsychoPy software package v.2022.2.5^43^ was used to program and conduct the experiment. A 23.8-inch wide-screen DELL monitor was used (resolution: 1920 × 1200) to present stimuli. Eye gaze was continuously recorded with a Tobii Pro Fusion eye-tracking system during the free-viewing task (with a sampling rate of 120 Hz). The eye-tracking system was fixed to the bottom of the monitor. The eye-tracking system was controlled by experimental presentation software through the ioHub event monitoring framework.

### Preprocessing of eye-tracking data

The data were preprocessed within PsychoPy. R.csv files containing the gaze data were saved after each experiment and exported through the ioHub Server with the relevant information about the variables and trials, including the start and end times of each single fixation on any area of interest. Areas of interest were defined by the relative position of presented pictures using PsychoPy’s normalized units. The left and right pictures were presented at -0.33, 0 and 0.33, 0, respectively.

Fixation durations were calculated for each area of interest by summing all fixation durations that exceeded the minimum threshold of 100 ms. Fixation durations that did not meet this minimum criterion were excluded from the analysis. Overall, 13.1% of all fixations were excluded. The mean exclusion rate did not vary as a function of emotion condition, *F*(5, 11747) = 0.36, *p* = .87, indicating comparable data quality across conditions.

### General procedure

Participants fulfilling our inclusion criteria were invited to the eye-tracking laboratory at the Department of Psychosomatic Medicine of the University of Leipzig. Participants’ visual acuity was tested with a Snellen eye chart. The individual sessions started with the completion of the questionnaires and tests under supervision of the experimenter. Then, participants sat in front of the eye-tracking monitor. They were asked to find a comfortable position and sit calmly throughout the task. All participants underwent the calibration procedure to make sure eye movements were recorded accurately. Hereafter, the free-viewing task was administered.

### Statistical analysis

The analysis of the eye-tracking data focused on fixation duration as an index of attention allocation. Total fixation duration was defined as the sum of all fixations on the emotional and neutral face respectively that had a minimum duration of 100 milliseconds. A total fixation duration bias score was calculated for each trial based on the difference between total fixation duration on the emotional face minus total fixation duration on the neutral face. A positive bias score means that on average total fixation duration on emotional faces was longer than on neutral faces. We calculated Spearman-Brown corrected split-half reliability with use of the split-half function from the split-half package (version 0.8.2) to assess reliability of our attention measure.

A repeated-measures linear mixed model approach (using the lmer function from the lme4 (version 1.1–36) package) was employed to investigate the effect of traumatic experiences in childhood (assessed via the CTQ subscale scores) on attention to emotional faces (operationalized as total fixation duration bias scores). To this end, we entered position and emotion as fixed effects into our model, alongside the scores of the CTQ subscales. A random intercept for participants was also included to account for initial differences between individuals and the correlation of the repeated measures acquired within each subject. Lastly, we integrated interaction effects between the CTQ subscales and each emotion condition to examine the differential effect of childhood maltreatment forms on attention allocation across different emotional qualities.

A second linear mixed model was fitted, which was identical to the first, except that we entered the CTQ total score instead of the individual CTQ subscale scores. By doing so, we examined the influence of overall childhood maltreatment severity on attention allocation to faces with different emotional qualities.

Statistical analyses were conducted in R (version 4.4.0) using RStudio (version 2024.04.0 + 735; RStudio, Inc.). Pairwise t-tests using the pairwise_t_test function from the rstatix (0.7.2) package were performed to examine the differences in total fixation duration between emotional and neutral faces. To obtain *F*-statistics, the finalized linear mixed models were subjected to Type III ANOVAs using the Anova function from the car package (version 3.1-3). The significance threshold was set at *p* ≤ .05. For significant main or interaction effects involving more than two levels (i.e., emotion), we planned to conduct post-hoc pairwise comparisons using the estimated marginal means (EMMs) via the emmeans function from the emmeans package (version 1.10.6). Significance thresholds were adjusted using the Tukey HSD method. Furthermore, Pearson correlations were computed between self-report and test measures. All CTQ subscale scores were mean-centered and examined for multicollinearity prior to model estimation. Correlations among the CTQ subscales were low to moderate (all *r* ≤ .40), indicating no evidence of problematic multicollinearity.

We performed simple slope analyses via the emtrends package that allowed to evaluate the specific directional effect of individual childhood maltreatment types on fixation duration bias across the different facial emotion conditions. As these simple slope analyses did not reach statistical significance, the detailed trend statistics are not presented.

## Results

### Relations of childhood maltreatment with emotion regulation, anxiety, depression, stress perception, alexithymia, and intelligence

Correlations between the CTQ total and subscale scores and the other psychometric variables are presented in Table 1. Significant positive correlations were observed between the CTQ total score and expressive suppression as well as depressive symptoms. The subscale emotional neglect correlated negatively with cognitive reappraisal and positively with expressive suppression, level of depressive symptoms, and general sense of stress. Emotional abuse showed only a correlation with depressive symptoms. Physical and sexual abuse were not related to emotion regulation style, trait anxiety, depressive symptoms, stress perception, alexithymia, or intelligence. Moreover, cognitive reappraisal was negatively related to depressive symptoms and perceived stress, whereas expressive suppression was positively associated with depressive symptoms and alexithymia. Intelligence was not correlated with any of the questionnaire measures (see Table 1 for details).

### Free-viewing task

#### Reliability of the free-viewing task

The reliability analysis showed high reliability coefficients for the bias scores (total fixation duration). Spearman-Brown corrected split-half reliability estimates (using 500 random splits) were above 0.9 for all emotion conditions (see Table 2 for reliability coefficients with 95% confidence intervals).

**Table 2:** Spearman-Brown and split-half reliability of fixation duration bias scores for emotion conditions (with 95% confidence intervals).

Face conditions	Spearman-Brown reliability	95% CI	Split-half reliability	95% CI
Happiness - neutral	0.93	[0.91, 0.95]	0.87	[0.83, 0.90]
Surprise - neutral	0.91	[0.88, 0.94]	0.84	[0.79, 0.88]
Anger - neutral	0.93	[0.90, 0.95]	0.87	[0.82, 0.90]
Disgust - neutral	0.95	[0.93, 0.96]	0.90	[0.87, 0.93]
Fear - neutral	0.94	[0.91, 0.96]	0.88	[0.84, 0.91]
Sadness - neutral	0.93	[0.90, 0.95]	0.86	[0.82, 0.90]

### Fixation duration bias

**Fig. 1:**
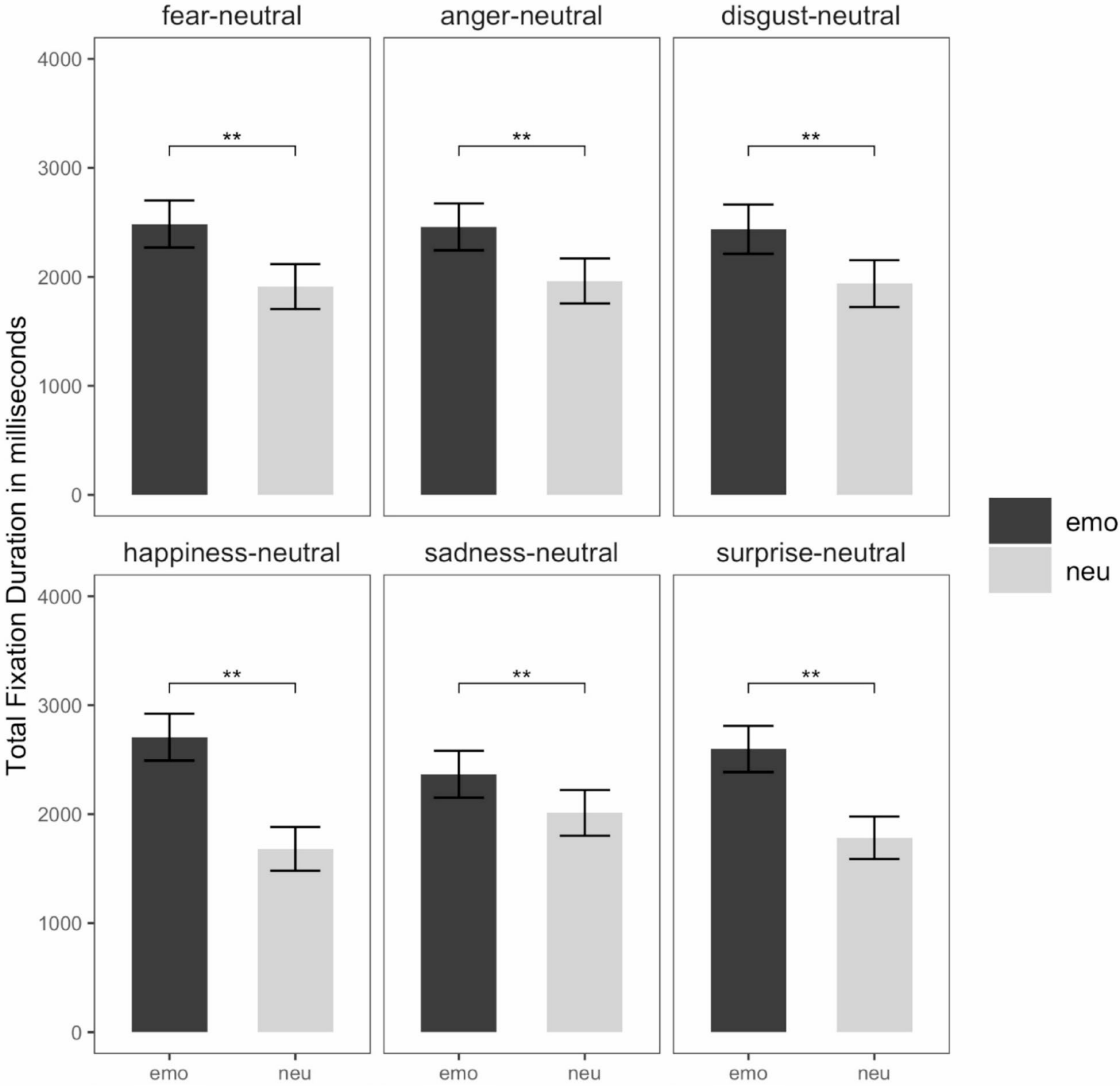
Fixation durations in milliseconds on the emotional (i.e., fearful, angry, disgusted, happy, sad, and surprised) and paired neutral facial expressions. Error bars denote standard error. ** indicates significant differences between fixation durations for emotional and paired neutral faces.

We present the mean total fixation durations for emotional and neutral faces in Fig. 1. To assess the difference between total fixation duration on the emotional and paired neutral faces we calculated paired sample *t*-tests. For all emotion categories, participants looked longer at the emotional facial expressions compared to the neutral facial expressions (see Table 3 for *t*-test results).

**Table 3:** Comparison of fixation duration for emotional and paired neutral faces (*t*-test results).

Face conditions	N1	N2	t	df	*p*
Happiness - neutral	1960	1960	26.10	1959	< 0.001
Surprise - neutral	1960	1960	21.40	1959	< 0.001
Anger - neutral	1960	1960	12.41	1959	< 0.001
Disgust - neutral	1960	1960	12.00	1959	< 0.001
Fear - neutral	1960	1960	14.47	1959	< 0.001
Sadness - neutral	1960	1960	8.92	1959	< 0.001

N1: Number of measurement values for fixation duration on specific emotional faces entered into the analysis.

N2: Number of measurement values for fixation duration on (paired) neutral faces entered into the analysis.

### The relationship of fixation duration bias with type and level of childhood maltreatment

According to the results of our linear mixed model including the CTQ subscales there was no significant main effect for childhood maltreatment type (i.e., physical, emotional, or sexual abuse or emotional neglect). Furthermore, no significant interaction effects between CTQ subscales and emotion category were observed. We found an effect of position indicating that emotional faces, which were shown on the left side, were viewed longer. Moreover, there was a main effect of emotion (see Table 4 for details). Post-hoc pairwise comparisons revealed significant differences in fixation duration bias across facial emotions. Happy faces elicited a significantly greater fixation duration bias than fearful (estimate = 443 ms, *p* < .001), angry (estimate = 542 ms, *p* < .001), disgusted (estimate = 534 ms, *p* < .001), sad (estimate = 679 ms, *p* < .001), and surprised faces (estimate = 229 ms, *p* < .001). Similarly, surprised faces elicited a significantly greater fixation duration bias than fearful (estimate = 214 ms, *p* < .001), angry (estimate = 313 ms, *p* < .001), disgusted (estimate = 306 ms, *p* < .001), and sad faces (estimate = 450 ms, *p* < .001). Furthermore, fixation duration bias was significantly greater for fearful faces compared to sad faces (estimate = 236 ms, *p* < .001), and for disgusted faces compared to sad faces (estimate = 145 ms, *p* = .043). No other pairwise differences between emotion conditions reached statistical significance.

**Table 4:** Linear mixed model main and interaction effects for attentional bias with childhood maltreatment types (model 1) and overall severity (model 2).

Predictor	df_num_	df_den_	F	*p*
Model 1: CTQ subscales				
Position of face (left, right)	1	11,636	4.64	0.031
Emotion category	5	11,636	6.90	< 0.001
Emotional abuse (CTQ)	1	122.73	0.02	0.889
Physical abuse (CTQ)	1	122.73	0.08	0.776
Sexual abuse (CTQ)	1	122.73	1.40	0.239
Emotional neglect (CTQ)	1	122.73	0.01	0.932
Emotional abuse x Emotion category	5	11,636	1.41	0.218
Physical abuse x Emotion category	5	11,636	0.14	0.984
Sexual abuse x Emotion category	5	11,636	2.17	0.055
Emotional neglect x Emotion category	5	11,636	1.68	0.135
Model 2: CTQ total score				
Position of face (left, right)	1	11,651	4.64	0.031
Emotion category	5	11,651	2.85	0.014
Overall maltreatment severity	1	127.06	0.14	0.707
Overall maltreatment severity x Emotion category	5	11,651	0.92	0.469

dfₙ_u_ₘ = numerator degrees of freedom; df_den_ = denominator degrees of freedom.

### The relationship of fixation duration bias with overall childhood maltreatment severity

The results of our second linear mixed model revealed no main effect for overall childhood maltreatment (as measured by the CTQ total score) and no interaction effect between overall childhood maltreatment and emotion category (see Table 4 for details). Consistent with our findings from the first model, there was a significant main effect of position indicating that emotional faces presented on the left side elicited longer fixation durations. There was also a significant main effect of emotion. Post-hoc comparisons for the emotion effect revealed the same pattern of results as observed in the first model.

## Discussion

By tracking eye movements during free viewing, we sought to determine how various types of childhood maltreatment are linked with spontaneous attention allocation to facial emotions in men. Our analysis focused on the duration of attention participants devoted to emotional compared to neutral faces and considered the severity of maltreatment experiences. To our knowledge, none of the previous studies on childhood maltreatment and attention to emotions has examined exclusively men (or boys). Investigating these associations specifically in male cohorts is justified by evidence that men experience unique patterns of childhood trauma^22,23^ and demonstrate diminished accuracy and attention during facial emotion processing relative to women^24,25^. Our results indicate neither a significant main effect of childhood maltreatment type, nor a significant interaction effect between childhood maltreatment type and emotion category. Moreover, no main effect or interaction involving overall childhood maltreatment severity was observed. Thus, the present findings reveal no significant relationship between childhood experiences of maltreatment (including emotional, sexual, and physical abuse, as well as emotional neglect) and visual attention to facial emotions in men. These data therefore do not support the hypothesis that early life trauma may lead to alterations in attentional processing of emotional expressions in men.

The present findings fail to support the hypothesized relationships. Our data do not provide confirmation for the hypotheses that at high levels of physical and emotional abuse attentional preference for positive compared to other emotional faces decreases and that at a severe level of emotional abuse a bias away from disgusted faces occurs. These assumptions were based on the results of a previous eye-tracking study that examined a sample of women^21^. Cross-study comparability is enhanced by the implementation of similar protocols for participant recruitment, experimental testing, and data processing across both investigations. The differences in the results of the two studies may point to sex-specific associations between childhood maltreatment types and attention allocation to emotional facial expressions in adulthood. Differences in sample size are unlikely to account for the observed null effects, as the current male sample (*N* = 98) is virtually identical in size to the previous female cohort (*N* = 100). Moreover, low reliability of the attentional bias indices can be ruled out as a cause of the observed null results in the male sample. When interpreting the null findings, it may also be informative to examine the means and standard deviations of the CTQ total and subscale scores in the present male sample compared to the previous female sample^21^. The CTQ total score is significantly higher for the women than for the men (58.30 (SD = 11.60) vs. 53.95 (SD = 13.06), *t* (196) = 2.45, *p* < .05). At the subscale level, women exhibited higher emotional abuse scores (15.62 (SD = 4.40) vs. 12.58 (SD = 4.76), *t* (196) = 4.67, *p* < .001) and higher emotional neglect scores (18.18 (SD = 3.91) vs. 16.83 (SD = 3.75), *t* (196) = 2.55, *p* < .05) than men. No significant differences between women and men are found for physical and sexual abuse (7.84 (SD = 3.95) vs. 8.68 (SD = 4.58), *t* (190.78) = -1.40, *p* = .16; 6.83 (SD = 3.82) vs. 6.34 (SD = 3.48), *t* (196) = 0.80, *p* = .42). Levene’s test indicates equal variances across groups, except for the CTQ physical abuse subscale (*p* < .01), which showed significantly greater variability in males than in females. The lower scores for emotional abuse and overall childhood maltreatment severity observed in the male sample compared to the female sample may have hampered the detection of associations with attentional biases. However, low variance in the CTQ scores within the male sample is unlikely to explain the observed null results.

The findings from our current male sample suggest that attention allocation to emotional faces may not be robustly altered by experiences of early life stress within this specific cohort. Regardless of their self-reported maltreatment history, the men in our sample demonstrated a general attentional bias toward emotional faces, particularly prioritizing happy over neutral expressions. These data raise the possibility that, in certain male populations, the neurocognitive mechanisms governing the attentional prioritization of facial happiness, surprise, anger, fear, sadness, and disgust might operate independently of a history of childhood maltreatment. Specifically, within the constraints of our paradigm, experiences of abuse did not appear to be accompanied by a statistically significant reduction in attentional preference for positive faces; similarly, emotional abuse was not observed to be reliably associated with a tendency to avoid hostile (disgusted) facial expressions. In men, the psychological sequelae of childhood maltreatment experiences manifest more prominently as externalizing behaviors such as substance use or conductive disorders^44–46^, which might not be associated with altered visual attention to other individual’s emotional facial expressions. In contrast, the psychological consequences of childhood maltreatment among girls more frequently manifest as internalizing disorders such as anxiety and depression in adulthood^47–49^. Depressed mood has been found to be linked to reduced attention allocation to positive facial expressions^50,51^. The present findings raise the question of whether - unlike in women - traumatic childhood experiences in men fail to impact attentional processes regarding emotional social signals in adulthood. Childhood maltreatment may manifest differently in adulthood depending on sex. This could include at least partially distinct vulnerabilities to psychopathology, as well as differences in how individuals attend to emotional stimuli. However, before firm conclusions on this can be drawn, further studies must replicate the observed sex-specific result patterns. The findings of the present study, along with those of Hoepfel et al.^21^, indicate that when investigating the relationships between childhood maltreatment and attention to emotions, it seems important to consider biological sex as a relevant variable.

In our investigation, a free-viewing task was employed to assess processes of attentional preference. The use of a free-viewing paradigm allowed us to examine self-generated, endogenously controlled gaze behavior^52^. We found high reliability coefficients for fixation duration bias scores across all emotion conditions. This is in line with previous eye-tracking research using the free-viewing task that reported fair to good reliability for indices of attentional bias based on fixation duration or dwell time^53^.

Several limitations of the present investigation warrant careful consideration. First, our participant sample was predominantly comprised of young, highly educated, white/Caucasian men with diverse histories of childhood maltreatment, which inherently restricts the external validity of our conclusions. Consequently, these findings may not generalize to individuals with lower educational attainment, older cohorts, or clinical populations. Beyond these sampling constraints, specific methodological considerations regarding our measurement approach must be addressed. A notable limitation of this study is the reliance on a retrospective, self-report instrument to assess experiences of childhood maltreatment. Although the CTQ represents a well-validated instrument for quantifying adverse childhood experiences, its scope is restricted to maltreatment within the familial environment and lacks precise temporal data regarding when the adversity occurred. Furthermore, self-report measures of early life trauma are subject to inherent methodological disadvantages and potential confounding factors, including memory-recall biases, state-dependent biases (such as those influenced by current negative affect), and systematic response biases (including minimization or exaggeration tendencies). Accordingly, it is imperative for future research to utilize prospective institutional records of childhood maltreatment alongside clinician-administered interview tools to determine whether the empirical patterns demonstrated herein can be robustly replicated. In addition to measurement constraints surrounding childhood trauma, limitations concerning the experimental stimuli and the selected eye-tracking metrics require discussion. Our investigation deliberately focused on the mechanisms underlying sustained attentional preferences rather than examining the early processes governing initial attentional orientation. Future research exploring the impact of childhood maltreatment on cognitive processing should specifically elucidate these early orienting mechanisms using reliable experimental paradigms. Whether novel computational methods for calculating attentional bias from the dot-probe task might offer valuable insights in this regard remains an open question^54^. Moreover, while our attentional bias metrics derived from raw differential values exhibited high reliability, they do not account for the absolute viewing duration directed toward the facial stimuli. For subsequent investigations, we therefore recommend implementing a proportional fixation-bias index of attention to effectively control for total viewing time. Finally, our design specifically evaluated attentional allocation toward expressions of happiness, surprise, anger, disgust, fear, and sadness. As a result, our conclusions cannot be generalized to the perception of other facial expressions or distinct basic emotions. It would be highly valuable for future investigations to incorporate facial expressions of additional emotions, such as shame and contempt, which frequently manifest as psychological sequelae of childhood trauma or as components of abusive behavioral dynamics^55,56^.

## Data Availability

The datasets used and/or analyzed during the current study are available from the corresponding author on reasonable request.

## Abbreviations

BDI-II: Beck depression inventory
CTQ: Childhood trauma questionnaire
ERQ: Emotion regulation questionnaire
EMM: Estimated marginal means
KDEF: Karolinska directed emotional faces
MWT-B: Mehrfachwahl-Wortschatz-Intelligenztest version B
PSS-10: Perceived stress scale
STAI: State-trait anxiety inventory
TAS-20: 20-item toronto-alexithymia scale
